# Presentation and Management of Granulomatous Mastitis in the United States: Results of an American Society of Breast Surgeons Registry Study

**DOI:** 10.1245/s10434-024-15714-x

**Published:** 2024-07-05

**Authors:** Nimmi S. Kapoor, Howon Ryu, Linda Smith, Jingjing Zou, Katrina Mitchell, Sarah L. Blair

**Affiliations:** 1 Division of Surgical Oncology, Department of Surgery, David Geffen School of Medicine at UCLA, Los Angeles, CA USA; 2 Herbert Wertheim School of Public Health and Human Longevity Science, University of California, San Diego, CA USA; 3 XRANM, Albuquerque, NM USA; 4 Department of Surgical Oncology, Ridley Tree Cancer Center, Santa Barbara, CA USA; 5 Division of Breast Surgery, Department of Surgery, University of California, San Diego, CA USA

**Keywords:** Granulomatous mastitis, Surgery, Intralesional steroids, Cosmesis

## Abstract

**Background:**

Granulomatous mastitis (GM) is a benign, chronic, inflammatory disease lacking clear treatment guidelines. The purpose of this American Society of Breast Surgeons (ASBrS) prospective, multisite registry was to characterize the presentation of GM and identify treatment strategies associated with symptom resolution and optimal cosmesis.

**Methods:**

ASBrS members entered data into a registry on patient demographics, treatment, symptoms, and cosmesis over a 1-year period. Initial symptoms were graded as mild, moderate, or severe. The Chi-square test and logistic regression were used to identify factors related to symptom improvement and cosmesis.

**Results:**

Overall, 112 patients with a mean age of 36 years were included. More patients were Hispanic (49.1%) and from the Southwest (41.1%), and management included observation (4.5%), medical (70.5%), surgical (5.4%), or combination treatment (19.6%). Immunosuppression was used in 83 patients (74.1%), including 43 patients who received intralesional steroid injections. Patients with severe symptoms were more likely to undergo surgical intervention compared with those with mild or moderate symptoms (21.4% vs. 0% and 7.5%, respectively; *p* = 0.004). Within 1 year, 85 patients (75.9%) experienced symptom improvement and/or resolution at a median of 3 months. Receipt of immunosuppressive therapy was predictive of improvement or resolution at 1 month (odds ratio 4.22; *p* = 0.045). One-year physician-assessed cosmesis was excellent or good for 20/35 patients (57.1%) and was not associated with type of treatment or symptom severity.

**Conclusion:**

Although GM can have a protracted course, the majority of patients in this registry resolved within 1 year, with good cosmetic result. Treatment with immunosuppression appears to be most beneficial, and a symptom-based algorithm may be helpful to guide treatment.

Granulomatous mastitis (GM) is an uncommon, benign, inflammatory disease of the breast that mimics malignancy and often has a chronic, relapsing course. Patients are most commonly non-Caucasian and present within 5 years of childbearing.^1^ Most patients present with a mass, swelling, fluid collections, and skin changes that can include ulceration and fistula, and imaging may reveal an irregular lesion mimicking malignancy.^1–3^ Diagnosis of GM is made by histopathologic examination of tissue with core needle biopsy or surgical excision. Hallmark pathologic features show non-caseating granulomas, lymphocytes, and multinucleated giant cells; however, numerous subtypes and classifications in the literature suggest a spectrum of this disease entity that can vary geographically and demographically.^1,4,5^ Etiology varies from infectious, traumatic, or idiopathic; nomenclature used to describe various forms of GM include idiopathic granulomatous mastitis (IGM), granulomatous mastitis (GM), idiopathic granulomatous lobular mastitis (IGLM), granulomatous lobular mastitis (GLM), and cystic neutrophilic granulomatous mastitis (CNGM).^6,7^

At initial presentation of GM, patients are often started on antimicrobials while further diagnostic imaging, cultures, and/or biopsies are pursued. In the past, surgical intervention was often the primary treatment for GM; however, many patients developed refractory or recurrent symptoms, including complex abscesses, sinus tracts, and recurrent episodes of inflammation. As a result, GM management has shifted from surgical to medical, utilizing a more systemic approach to both limit morbidity and expedite resolution. However, for those cases that are refractory to initial, expectant, or limited intervention, a long-term course of antimicrobials or immunosuppressive therapy such as prednisolone or methotrexate may be required. Prolonged courses of treatment with either antimicrobials or corticosteroids can lead to significant morbidity, whereas surgical intervention can also lead to wound complications, fistula formation, and poor cosmesis.^2,8,9^ More recent data show benefit of intralesional steroid injection or other immune-modulating agents in the management of GM.^7,10,11^

Ideal treatment duration, incidence of recurrence, and surgical indications for GM remain unclear. Furthermore, most large studies of patients with GM have been conducted outside of North America or within a limited patient demographic, preventing broad generalization of optimal treatment for a North American-based population.^1,9,12^ The purpose of this registry study was to collect prospective multi-institutional data from members of the American Society of Breast Surgeons (ASBrS), of the most common presentation and management of GM over a large geographic and demographic distribution. Patients in this registry who benefit from medical versus surgical management will be identified and further characterized based on symptom presentation. In addition, treatment strategies that result in shortest duration of symptoms and optimal cosmesis from the registry will be used to develop an algorithm to guide treatment.

## Methods

Surgeons signed an attestation document to prospectively enter information into the Idiopathic Granulomatous Mastitis (IGM) Registry created in the Mastery of Breast Surgery^®^ database for patients undergoing treatment of GM between July 2022 and August 2023. At the time of registry creation, IGM was a recognized term in the United States, however others have recommended use of more descriptive terminolology.^5,6^ For clarity, we subsequently chose to use ‘granulomatous mastitis’ as it encompasses all entities by histopathology rather than disease etiology. No monetary compensation for data entry was offered. Participation was voluntary. Recruitment occurred through an ASBrS newsletter and member emails. The principal investigator’s Institutional Review Board (IRB; #807448), ‘Treatment patterns in granulomatis mastitis’, the ASBrS Research Committee, and study co-investigators approved the study design. Surgeons who participated in the registry chose which of their patients to include in the database. Patients entered with incomplete baseline data on treatment or symptoms, or patients without any follow-up recorded were excluded. Reminders to complete data entries were sent out via email directly to participating surgeons on three separate occasions over the course of the registry.

Data on patient demographics, age, race, postpartum status, disease severity, laterality, method of diagnosis, and bacterial cultures were collected. Initial symptom severity was graded in one of three categories: mild, involving <10% of the breast; moderate, involving 10–25% of the breast; or severe, involving >25% of the breast.

### Treatment and Symptoms

Initial treatment included the first method of GM management by the ASBrS member treating the patient and did not include treatments delivered prior to presentation.

Medical intervention included use of non-steroidal anti-inflammatories, antibiotics, steroids and other immune-modulating agents, and topical medications. Intralesional steroid injection was included as a medical intervention even if aspiration was included. Steroid treatment category included topical and oral steroids as well as other immune-modulating agents, including methotrexate and azathioprine.

Surgical intervention included needle aspiration if performed without steroid injection, incision and drainage, and excision. All treatment was defined as interventions over the course of a patient’s follow-up within the registry and included categories of observation, medical, surgical, or both medical and surgical. Changes in treatment were captured at 1, 3, 6, and 12 months where applicable.

Symptom follow-up was reported at 1, 3, 6, and 12 months and was determined by the reporting physician from routine clinic visits and recorded by the physician into the database. Patients were included in the analysis if at least one symptom follow-up was reported. Categories of symptoms at follow-up included worse, stable, improved, or resolved. For study analysis, categories of worsening and stable (worse/stable) were combined and categories of improved and resolved (improved/resolved) were combined.

In addition, data on cosmesis at 1, 3, and 6 months were reported by the surgeon and included categories of worsened, stable, or improved. Final 1-year cosmesis was captured as poor, fair, good, or excellent^13^ and was defined as follows:

*Poor:* Marked change in the appearance of the affected breast involving >25% of the breast tissue. The skin changes may be obvious and may detract from the appearance of the breast. Severe scarring, fistulas, and thickening of the breast, which clearly alter the appearance of the breast, may be found.

*Fair:* Obvious difference in the size and shape of the affected breast. This change involves <25% of the breast. There can be moderate thickening or scar tissue of the skin and the breast.

*Good:* There is a slight difference in the size or shape of the treated breast compared with the original appearance of the treated breast. The thickening or scar tissue within the breast causes only a mild change in the shape or size.

*Excellent:* No visible changes due to treatment

### Algorithm Development

Data from the registry will be used to extrapolate type and timing of treatment interventions that are associated with both symptom resolution and optimal cosmesis. Patient factors, disease severity, treatment, and combination treatment over the course of the registry were considered.

### Statistical Methods

The Chi-square test, two-sample t-test, and Fisher’s exact test were used for univariate analyses to identify covariates associated with improved/resolved symptoms compared with patients with worse/stable symptoms. Logistic regression analysis was then performed to identify treatments associated with 1-month improved/resolved symptoms. Covariates and potential confounders, including age, race, demographics, severity, extent of disease at presentation, postpartum status, bacterial culture result, and intial treatment were adjusted in the model. Similarly, the Chi-square test, two-sample t-test, Fisher’s exact test, and logistic regression were used to evaluate data on cosmesis. All statistical analysis was conducted using R 4.3.1 software (The R Foundation for Statistical Computing, Vienna, Austria).

## Results

### Registry Cohort

Overall, 112 patients, entered by 45 surgeons, had sufficient data for inclusion (Table 1). Median follow-up for the whole cohort was 3 months (range 1–12 months). Mean patient age was 36 years (range 19–64), most patients were either Hispanic (49.1%) or Caucasian (21.4%), and more patients were from the Southwest (41.1%). Most patients had unilateral disease presentation and underwent core needle biopsy for confirmation of diagnosis. Sixty patients (53.6%) had bacterial cultures performed and 26 of these patients (43.3%) had positive cultures, including 20/26 patients (76.9%) with bacterial growth of common skin flora of corynebacterium and other diptheroid species. Approximately half of the patients presented with moderate symptoms (47.3%), while the remainder of the patients presented with either mild (27.7%) or severe (25.0%) symptoms.

**Table 1:** Clinical and treatment characteristics of all patients and comparison by one-year symptom improvement/resolution (I/R)

	Overall	One-Year I/R		*p*-value
		No	Yes	
Number of Patients	112	27	85	
Patient Age, Mean (SD)	36.0 (8.4)	33.1 (6.7)	36.9 (8.7)	0.041
Race/Ethnicity (%)				0.731
African American	15 (13.4)	4 (14.8)	11 (12.9)	
Arabic or Middle Eastern	3 (2.7)	1 (3.7)	2 (2.4)	
Asian or Pacific Islander	10 (8.9)	1 (3.7)	9 (10.6)	
Caucasian	24 (21.4)	4 (14.8)	20 (23.5)	
Hispanic	55 (49.1)	16 (59.3)	39 (45.9)	
Native American Indian	5 (4.5)	1 (3.7)	4 (4.7)	
Demographic (%)				0.214
Midwest	11 (9.8)	1 (3.7)	10 (11.8)	
Northeast	23 (20.5)	6 (22.2)	17 (20.0)	
Northwest	7 (6.2)	4 (14.8)	3 (3.5)	
Southeast	25 (22.3)	5 (18.5)	20 (23.5)	
Southwest	46 (41.1)	11 (40.7)	35 (41.2)	
Method of Diagnosis (%)				0.203
Clinical examination only	13 (11.6)	3 (11.1)	10 (11.8)	
Core biopsy	90 (80.4)	24 (88.9)	66 (77.6)	
Surgical	9 (8.0)	0 (0.0)	9 (10.6)	
Extent (%)				0.508
Mild	31 (27.7)	7 (25.9)	24 (28.2)	
Moderate	53 (47.3)	11 (40.7)	42 (49.4)	
Severe	28 (25.0)	9 (33.3)	19 (22.4)	
Laterality (%)				0.687
Bilateral	8 (7.1)	1 (3.7)	7 (8.2)	
Unilateral	101 (90.2)	25 (92.6)	76 (89.4)	
NA	3 (2.7)	1 (3.7)	2 (2.4)	
Postpartum (%)				0.14
No	91 (81.2)	19 (70.4)	72 (84.7)	
Yes	16 (14.3)	7 (25.9)	9 (10.6)	
NA	5 (4.5)	1 (3.7)	4 (4.7)	
Wound Culture Performed (%)				0.1
No	49 (43.8)	7 (25.9)	42 (49.4)	
Yes	60 (53.6)	19 (70.4)	41 (48.2)	
NA	3 (2.7)	1 (3.7)	2 (2.4)	
Growth Found on Culture (%)				0.198
NA	49 (43.8)	8 (29.6)	41 (48.2)	
Negative	34 (30.4)	10 (37.0)	24 (28.2)	
Positive	26 (23.2)	9 (33.3)	17 (20.0)	
Unknown	3 (2.7)	0 (0.0)	3 (3.5)	
Change in Treatment (%)				0.144
No	46 (41.1)	7 (25.9)	39 (45.9)	
Yes	65 (58.0)	20 (74.1)	45 (52.9)	
NA	1 (0.9)	0 (0.0)	1 (1.2)	
All Treatment (%)				0.089
Observation	5 (4.5)	0 (0.0)	5 (5.9)	
Medical Only	79 (70.5)	24 (88.9)	55 (64.7)	
Surgical	6 (5.4)	0 (0.0)	6 (7.1)	
Combined Medical and Surgical	22 (19.6)	3 (11.1)	19 (22.4)	

### Treatment

Treatment intervention included medical (70.5%), surgical (5.4%), or a combination of medical and surgical interventions (19.6%); 5 patients (4.5%) underwent observation alone. While a majority of patients (*n* = 73, 65.2%) were started on antibiotics and/or non-steroidal anti-inflammatories, by 1 month only 12 patients (10.7%) were reported to be taking these agents, and by 3 months, only 2 patients (18.2%) were still taking them. Of the entire cohort, 74.1% (*n* = 83) received some form of steroid or other immunosuppressive therapy, including 57 patients (50.9%) who were taking oral steroids, 43 patients (38.4%) who were taking intralesional steroids, 16 patients (14.2%) who received methotrexate, and 3 patients (2.7%) who were taking azathioprine. Of the 83 patients who received immunosuppressive treatment, 17/83 (20.5%) received both intralesional steroids and oral immunosuppression, while 26/83 (31.3%) received intralesional steroids without oral immunosuppression and 40/83 (48.2%) had oral immunosuppression without intralesional steroids. Patients who presented with severe symptoms were more likely to undergo initial surgical intervention compared with those with mild or moderate symptoms (21.4% vs. 0% and 7.5%, respectively; *p* = 0.004) (Table 2).

**Table 2:** Initial treatment by severity of symptom presentation

	All	Observation	Medical Treatment	Immunosuppressive Therapy	Surgical Intervention	*p*-value
Presenting Symptoms, n (%)	112	43	45	14	10	0.004
Mild	31	15 (34.9)	8 (17.8)	8 (57.1)	0 (0.0)	
Moderate	53	19 (44.2)	24 (53.3)	6 (42.9)	4 (40.0)	
Severe	28	9 (20.9)	13 (28.9)	0 (0.0)	6 (60.0)	

### Relapse and Resolution

Of 67 patients who experienced stable, improved, or resolved symptoms during the registry period, 17/67 (25.3%) had relapsing and worsened symptoms at 3 months (*n* = 13/67, 19.4%), 6 months (*n* = 3/67, 4.5%), or 12 months (*n* = 1/67,1.5%). Likewise, over the duration of the registry timeline, more than half of the patients (58.0%) underwent a change in treatment. Within 1 year, 85 patients (75.9%) experienced improved/resolved symptoms without further relapse at a median time of 3 months. Of those 85 patients, 30 experienced improved/resolved symptoms at follow-up of 1 month (*n* = 30/85, 35.3%), 3 months (*n* = 24/85, 28.2%), 6 months (*n* = 19/85, 22.4%), or 12 months (*n* = 12/85, 14.1%).

Of the 30 patients (26.8%) who experienced improved/resolved symptoms within 1 month without a relapse, 20/30 (66.7%) had medical intervention alone, including 8/20 (40%) who received intralesional steroids. On logistic regression analysis (Table 3), receipt of immunosuppressive therapy was predictive of improved/resolved symptoms at 1 month (odds ratio [OR] 4.225; *p* = 0.045). Also on logistic regression, race/ethnicity was associated with 1-month improved/resolved symptoms; groups other than Caucasian or Middle Eastern were more likely to experience 1-month improved/resolved symptoms (Table 3).

**Table 3:** Logistic regression analysis of one-month improvement/resolution of symptoms.

	Odds ratio	95% Confidence interval	*p*-value
Patient Age	1.03	(0.96, 1.12)	0.411
Race/Ethnicity			
African American	37.04	(1.87, 2368.47)	0.039
Arabic or Middle Eastern	11.18	(0.19, 1145.96)	0.244
Asian or Pacific Islander	127.49	(6.15, 8316.53)	0.006
Caucasian	Ref	Ref	Ref
Hispanic	37.19	(2.67, 1972.39)	0.025
Native American Indian	188.48	(6.59, 15568.42)	0.006
Demographic			
Midwest	8.71	(0.57, 291.49)	0.144
Northeast	1.08	(0.18, 6.13)	0.926
Northwest	2.82	(0.21, 36.60)	0.413
Southeast	0.49	(0.09, 2.39)	0.39
Southwest	Ref	Ref	Ref
Extent			
Mild	Ref	Ref	Ref
Moderate	1.40	(0.27, 8.17)	0.694
Severe	1.18	(0.20, 7.69)	0.857
Postpartum (Ref=no)	0.39	(0.03, 2.60)	0.377
Wound Culture			
Negative	Ref	Ref	Ref
Positive	4.16	(0.71, 29.43)	0.125
No Culture	4.48	(1.04, 26.52)	0.062
Unknown	0	(0, Inf)	0.993
Initial Treatment			
Surgery	3.14	(0.22, 38.59)	0.371
Immunosuppression	4.22	(1.1, 18.99)	0.045
Observation	2.64	(0.33, 20.80)	0.348
Medical (non-steroid)	Ref	Ref	Ref

*Ref* reference group, *Inf* infinity.

Patients with 1-year improved/resolved symptoms were slightly older than patients with worse/stable symptoms on univariate analysis using the two-sample t-test (36.9 vs. 33.1 years; *p* = 0.041). However, unlike 1-month symptom resolution, no other variables were associated with 1-year improved/resolved symptoms, including patient race, demographics, disease severity, positive bacterial culture, or treatment. On logistic regression, patients who received a combination of medical and surgical treatment were more likely to have improved/resolved symptoms within 1 year than those who only received medical treatment (OR 11.22, *p =* 0.014).

Within the registry time, more patients receiving oral immunosuppression alone experienced improved/resolved symptoms (*n* = 31/40, 77.5%) compared with intralesional steroids alone (*n* = 18/26, 69.2%) or both intralesional steroids and oral immunosuppression (*n* = 12/17, 70.6%), however this was not found to be significant (*p* = 0.5).

### Cosmesis

**Fig. 1:**
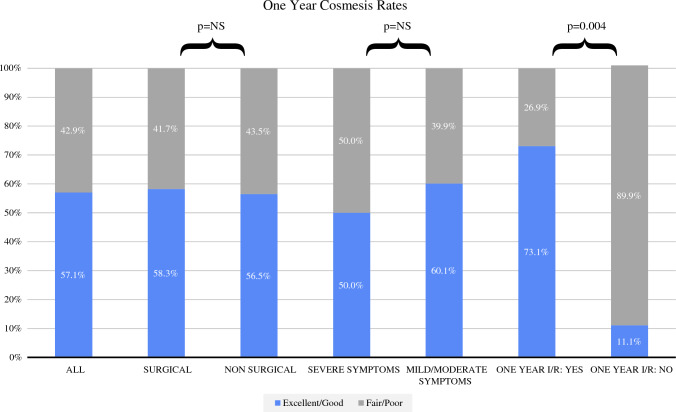
One-year cosmesis rates for 35 patients. ‘Surgical’ includes all patients with any surgical intervention. *I/R* improvement/resolution, *NS* non-significant

Within the registry, 92% of patients (*n* = 103) had an entry for cosmesis data in at least one time point, and 91 patients (88.3%) had multiple timepoint recordings for cosmesis. Of these 91 patients, 17.6% (*n* = 16) were described as having worsened cosmesis after having improved or stable cosmesis. Final 1-year cosmesis was recorded for 35 patients, with 20 patients (57.1%) having excellent (*n* = 6/20) or good (*n* = 14/20) cosmesis and the remainder (42.9%) having fair (*n* = 11/15) or poor (*n* = 4/15) cosmesis. Of the patients with 1-year cosmesis recorded (Fig. 1), patients with improved/resolved symptoms at 1 year were more likely to have excellent or good (excellent/good) cosmesis compared with patients who did not achieve 1-year improved/resolved symptoms (73.1% vs. 11.1%; *p* = 0.004). Patients who had surgical intervention, with or without medical intervention, were as likely to have excellent or good cosmesis at 1 year as patients who had non-surgical intervention (58.3% and 56.5%, respectively; *p* = 1.0). Similarly, 1-year cosmesis was reported to be excellent or good for 50% of patients who presented with severe GM symptoms and 60.9% of patients with mild or moderate GM symptoms at presentation (*p* = 0.72).

### Algorithm

**Fig. 2:**
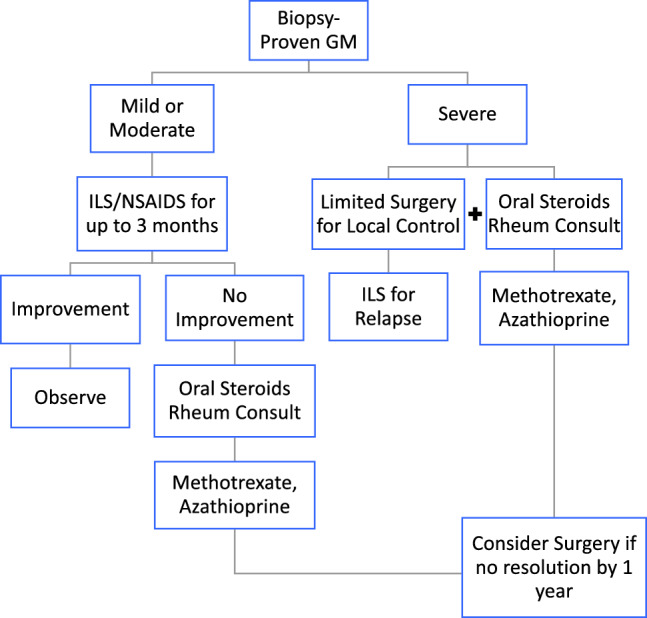
Algorithm for management of biopsy-proven granulomatous mastitis. *GM* granulomatous mastitis, *ILS* intralesional steroids, *NSAIDs* non-steroidal anti-inflammatory drugs, *Rheum* rheumatology

Based on the findings of this registry, the algorithm shown in Fig. 2 has been developed to help guide treating clinicians in the management of biopsy-proven GM. Since patients in this registry with severe symptoms were significantly more likely to undergo surgical intervention than those with mild or moderate symptoms, the algorithm was developed with consideration for symptom presentation. For patients presenting with mild or moderate symptoms, intralesional steroids with non-steroidal anti-inflammatories are recommended for up to 3 months as necessary. If no improvement or relapsing symptoms occur, consideration of oral immunosuppression and concurrent rheumatology consult is recommended when available. In severe cases, limited surgical intervention may be necessary initially for symptom relief or management of local wound concerns, while concurrent oral steroids are initiated and rheumatology is consulted. For relapsing or persistent symptoms, use of methotrexate or azathioprine and intralesional steroids should be utilized. Furthermore, since there was no significant cosmetic difference in patients who did ultimately require surgical management compared with non-surgical management, and since combination treatment with medical and surgical intervention was associated with 1-year improved/resolved symptoms, consideration for surgery is included within the algorithm, such that if persistent or relapsing symptoms are still present by 1 year, surgical management should be considered. In addition, while many patients may be started on antibiotics while awaiting cultures and biopsy results, most bacterial cultures in the setting of biopsy-proven GM either do not show any bacterial growth or grow bacteria that are related to common skin flora and contaminants. For the small percentage of patients with both biopsy-proven GM and an active bacterial infection, concurrent use of antibiotics should be considered alongside the algorithm guidelines.

## Discussion

GM is a chronic inflammatory condition with a relapsing course that can require multimodality treatment. In this ASBrS registry, we found that many demographics were represented within the database, and a majority were Hispanic and from the Southwest. While most patients had medical treatment, patients who presented with severe symptoms were most likely to undergo surgical intervention.

Some of the largest studies in the literature evaluating the role of surgery in GM are non-US-based.^2,9,12^ In a Turkish study of 720 patients, 64% of patients had surgical intervention, with or without medical intervention, including wide local excision, abscess drainage, and mastectomy.^9^ While relapse or recurrence was seen in 20% of patients, no difference in relapse rate was noted between patients who underwent surgical intervention, medical intervention, or combination therapy. In a Thai study, surgical intervention was noted to have the shortest time to healing in a group of 44 patients with GM, however 52% of patients developed wound complications.^2^ In contrast, a retrospective study of 73 patients with GM in India found that compared with observation alone, patients undergoing surgical management were less likely to have recurrence.^8^

Recurrence of GM has been a focus of several meta-analyses. In one meta-analysis of 12 studies including 559 GM patients, there was no difference in the recurrence risk ratio (RR) between patients who had surgery only compared with patients who received steroids only. However, the ‘risk difference’ in the steroid-only group was higher than in the steroids + surgery group, leading the authors to suggest combination therapy may be more effective.^12^ In a more recent larger meta-analysis of 71 studies including 4735 patients, recurrence rates were seen among 17.2% of patients, with a non-significant difference in relapse seen between patients who received surgical (22.5%), immunosuppressive (14.7%), or combined (14.9%) treatment.^14^

In our registry, 25.3% of patients experienced relapsing symptoms and over half underwent a change in treatment. Patients who received immunosuppression were more likely to have improved/resolved symptoms by 1 month, and combination medical and surgical therapy was predictive of improved/resolved symptoms by 1 year. A notable strength of this study is the prospective nature of data collection, which could have led to improved identification of relapses compared with most other GM studies, i.e. retrospective chart reviews.

Immunosuppression with oral steroids in the treatment of GM has long been described and has become the focus of primary medical management of GM.^15^ In this registry, 74.1% of patients received immunosuppression and most received this as oral immunosuppression alone, while some received intralesional steroids in addition to, or instead of, oral therapy. Intralesional steroids have only recently been described and studied as a treatment modality for GM. Most studies report using 20 mg/cm^3^ of triamcinolone acetonide (or similar steroid) either alone, with saline, or with lidocaine then injected into the center of the lesion, or at multiple sites depending on the volume involved, and repeated as often as twice weekly or as infrequently as every 4 weeks as needed.^11,16,17^ Several studies have compared systemic oral immunosuppression versus intralesional steroids, and have shown equal or superior benefit of intralesional steroids over oral immunosuppression with fewer systemic adverse effects.^11,16,17^ A 2024 meta-analysis focused on the role of local therapy in GM, including both intralesional steroids and topical steroid ointment, and compared local therapy with systemic therapy and surgical management.^16^ In this meta-analysis, 8 trials with 613 patients were included; 7 of the trials were randomized controlled trials from Turkey (*n* = 2) or China (*n* = 5), and one was a prospective registry from Turkey. Compared with systemic therapy or surgical intervention, intralesional steroids demonstrated an improved response rate (RR 1.35), lower adverse effects (RR 0.24), and no difference in recurrence rates.

Similarly, in our registry, in part due to small numbers, no difference was seen in 1-year improved/resolved symptoms between patients who received oral immunosuppression alone, intralesional steroids alone, or a combination of oral immunosuppression + intralesional steroids. As such, to avoid the significant morbidity and systemic adverse effects of oral immunosuppression that can include weight gain, hyperglycemia, osteoporosis, and worsening infections,^18,19^ first-line therapy with intralesional steroids is recommended for mild or moderate symptoms requiring intervention.

Additional studies have shown the benefit of other immune-modulating options such as methotrexate and azathioprine in protracted courses of GM, especially in patients who are intolerant, resistant, or incapable of taking oral steroids, with relapse-free remission ranging from 58 to 100%.^19–22^ While these medications are traditionally prescribed and managed by rheumatologists, surgeons treating patients with GM should be aware of other immune-modulating agents that might be useful in the management of patients with protracted GM symptoms or severe adverse effects from prolonged use of oral steroids.

Recent studies have analyzed treatments for GM to establish an optimal treatment modality and algorithm to limit symptom duration and relapse while minimizing treatment toxicity.^6,9,23,24^ In contrast to our prospective registry, a majority of the studies assessing optimal treatment are retrospective in nature and very few studies are randomized. Some studies have shown that observation alone may be sufficient,^25,26^ and likewise in our registry, 5 patients (5.9%) were able to experience improved/resolved symptoms with observation alone. Alternatively, a majority of studies have suggested that GM management should be adjusted based on clinical factors and symptom severity.^23,24^ A 2024 meta-analysis of 65 studies assessing optimal treatment of GM found recurrence rates of 4% with combination therapy of steroids and methotrexate, 7% with steroids and surgery, and up to 65% recurrence with drainage alone.^23^ Still, other studies have suggested that immune-modulating agents alone may be effective in the treatment of GM.^7,20,22^ In our study, 16 patients (14.3%) also received methotrexate in combination with steroid therapy. We found combination medical therapy and some form of surgical intervention to be most effective in achieving 1-year improved/resolved symptoms, consistent with other studies.^14,18,24^ Larger studies evaluating the degree of surgical intervention will be useful to understand the role of surgery in the management of GM.

### Cosmesis

To our knowledge, this is one of the first studies of GM to capture prospective data on cosmesis outcomes over a 1-year period. Similar to symptom relapse, cosmesis was seen to improve and then worsen over the study period for 17.6% of patients. While cosmesis was clinician-reported, only 57.1% reported excellent or good patient cosmesis, indicating an opportunity for improving patient outcomes. Overall, 11.1% of patients who did not have improved/resolved symptoms by 1 year were unlikely to have excellent or good cosmesis at 1 year, compared with 73.1% of patients who did achieve improved/resolved symptoms within 1 year (*p* = 0.004). Interestingly, half of the patients who presented with severe symptoms had excellent or good 1-year cosmesis and were as likely as those with mild or moderate symptoms to have excellent or good 1-year cosmesis. This may be useful information to convey to patients with acute severe symptoms who may be concerned with their cosmetic outcome.^18^ Likewise, surgical intervention did not dictate worse cosmetic outcomes. Smaller studies have described extensive breast scarring and deformation after surgical excision in comparison with conservative or medical management.^27^ These differences in results are likely due to the extent of surgical intervention, with wide excision more likely to leave residual scar and defect than smaller drainage procedures.

### Limitations

Registry studies such as this are limited by reliance on contributing participants to accurately enter data. Patients who are lost to follow-up, or datapoints that are missing, can affect study results. Patient symptom and treatment variables prior to ASBrS member consultation could impact overall findings as well as timeline of symptom resolution. Nonetheless, these data are generalizable to how patients would present to members of the ASBrS. Additionally, extent of surgical management, i.e. abscess drainage and wide excision versus a larger procedure, was not individually captured, thus preventing broad generalizations on the recommended type of surgical interventions in the proposed algorithm. Nonetheless, we recommend limited surgical intervention when necessary due to the potential for wound complications and delayed healing noted in other studies.^27^ Similarly, frequency of either oral immunosuppression or intralesional steroid administration and dosage of steroid delivered were not captured to provide specific recommendations on the use of intralesional steroids in the algorithm. It is also important to note that cosmesis data were physician-assessed rather than patient-assesed, and in this registry, only a fraction of patients had data on 1-year cosmesis. Lastly, since many patients did not have further registry follow-up after symptom resolution, median follow-up was 3 months, and the limited timeline of this registry prevents the capture of recurrent episodes or changes in cosmesis beyond 1-year follow-up.

## Conclusion

Within the confines of this US-based registry study, we found that most patients with GM will have symptom improvement within 1 year but fewer than one-third will experience symptom improvement or resolution by 1 month. Immunosuppressive therapy is associated with fastest symptom resolution and is recommended as first-line intervention. Intralesional steroids is the preferred initial treatment over oral immunosuppression in mild or moderate cases, while oral immunosuppression with or without limited surgical intervention may be necessary in more severe cases.
